# An inherently infinite-dimensional quantum correlation

**DOI:** 10.1038/s41467-020-17077-9

**Published:** 2020-07-03

**Authors:** Andrea Coladangelo, Jalex Stark

**Affiliations:** Computing and Mathematical Sciences, Caltech, USA

**Keywords:** Quantum information, Theoretical physics

## Abstract

Bell’s theorem, a landmark result in the foundations of physics, establishes that quantum mechanics is a non-local theory. It asserts, in particular, that two spatially separated, but entangled, quantum systems can be correlated in a way that cannot be mimicked by classical systems. A direct operational consequence of Bell’s theorem is the existence of statistical tests which can detect the presence of entanglement. Remarkably, certain correlations not only witness entanglement, but they give quantitative bounds on the minimum dimension of quantum systems attaining them. In this work, we show that there exists a correlation which is not attainable by quantum systems of any arbitrary finite dimension, but is attained exclusively by infinite-dimensional quantum systems (such as infinite-level systems arising from quantum harmonic oscillators). This answers the long-standing open question about the existence of a finite correlation witnessing infinite entanglement.

## Abstract (web summary)

The existence of nonlocal correlations which cannot be attained exactly by finite-dimensional systems, but can be attained by infinite-dimensional ones, has been the subject of several theoretical efforts. Here, Coladangelo and Stark exhibit such a correlation, in a form that requires only two players.

## Introduction

Consider two spatially isolated quantum systems. For the sake of exposition, imagine that the first system is held by “Alice”, and the second by “Bob”. Consider a scenario in which an experimentalist is testing the two systems by choosing measurement settings and recording measurement outcomes. We will think of the experimentalist as a “referee” who sends classical questions to Alice and Bob, and receives answers from each of them. The behaviour of Alice and Bob is captured by the joint distribution of their answers as a function of their questions. We refer to this data as a bipartite correlation. It is natural to ask what can be learnt from a correlation, without assuming anything other than the spatial separation of Alice and Bob. Some correlations can be realized by classical parties (i.e. without any quantum entanglement, but possibly using a shared classical resource such as identical uniformly distributed strings). However, Bell’s theorem shows that there exist correlations which require entanglement to be realized^1^. The most well-studied example of this phenomenon is the CHSH game^2^. In the CHSH game, the referee asks questions *x*, *y* ∈ {0, 1} to Alice and Bob respectively, who reply with answers *a*, *b* ∈ {0, 1}. They win if *a* ⊕ *b* = *xy*. It is easy to see that, if the questions are sampled uniformly at random, the optimal winning probability without entanglement is 3/4. However, by sharing a maximally entangled pair of qubits and performing the appropriate measurements they can win with probability ${\cos }^{2}\frac{\pi }{8}\approx 0.85$. From an operational perspective, such a game gives a statistical test that can detect the presence of entanglement: any pair of systems which can win the CHSH game with probability >3/4 must possess entanglement. It is natural to wonder if anything stronger can be inferred solely from the correlation of two systems. Remarkably, certain correlations not only witness the presence of entanglement, but they provide quantitative bounds on the minimum dimension of quantum systems that attain them. Such correlations are referred to as dimension witnesses^3^, and have important applications in quantum cryptography, where they are used to certify that potentially untrusted quantum systems possess high-dimensional entanglement^4–8^. In this work, we push even further the investigation of what can be learnt from a correlation by addressing the question of whether there exists a correlation which witnesses infinite-dimensional entanglement. If it existed, one could view such correlation as a finite classical fingerprint of an infinite-dimensional quantum state.

Beyond potential applications to dimension certification, this question has fundamental importance within the theory of entanglement. From a foundational perspective, one of the most natural questions one can ask about a correlation is “in which models of physics can the correlation be realized?” The example of the CHSH game shows that while certain correlations can be realized in classical physics, others require quantum resources. Taking this a step further, different models of quantum mechanics allow Alice and Bob to achieve different sets of correlations, and characterizing the relationship between these sets is a long-standing problem, with implications on the way we model entanglement, and quantum mechanical resources more generally, and with potential repercussions on practical experiments (see the “Discussion” section).

We say that a correlation is in the set of quantum correlations ${{\mathcal{C}}}_{q}$ if there is a state $\left|\psi \right\rangle \in {{\mathcal{H}}}_{A}\otimes {{\mathcal{H}}}_{B}$, where ${{\mathcal{H}}}_{A}$ and ${{\mathcal{H}}}_{B}$ are finite-dimensional Hilbert spaces, and projective measurements $\left\{{A}_{x}^{a}\right\},\left\{{B}_{y}^{b}\right\}$ on ${{\mathcal{H}}}_{A}$ and ${{\mathcal{H}}}_{B}$ respectively, so that

1 $$p(a,b| x,y)=\left\langle \psi | {A}_{x}^{a}\otimes {B}_{y}^{b}| \psi \right\rangle ,$$

where *p*(*a*, *b*∣*x*, *y*) is the probability that Alice answers *a* and Bob answers *b*, given that Alice was asked question *x* and Bob was asked question *y*. We will refer collectively to the joint state of Alice and Bob and their measurements as their quantum strategy. In quantum computing, this is the typical way of modeling spatially separated quantum systems: as finite-dimensional Hilbert spaces in tensor product. If one allows the quantum systems to be infinite-dimensional (one can think for example of two infinite-level systems arising from quantum harmonic oscillators), the resulting set is denoted as $\scriptstyle{{\mathcal{C}}}_{qs}$. Notice that, of course, $\scriptstyle {{\mathcal{C}}}_{q}\subseteq {{\mathcal{C}}}_{qs}$. The question of whether this inclusion is strict, i.e., whether the two sets are equal or not, was first posed by Tsirelson in 1993^9^ (amongst other open questions), and has been unresolved since then.

In this work, we settle this long-standing open question by asserting that ${{\mathcal{C}}}_{q}\;\ne\;{{\mathcal{C}}}_{qs}$. In particular, we describe an explicit correlation on five questions per party and three answers per party, which can be attained exactly in infinite dimensions, and we show that it cannot be attained in finite dimensions. In other words, we provide an example of an inherently infinite-dimensional quantum correlation. What is particularly striking about this correlation is that the corresponding Bell scenario is finite (it involves a finite number of measurement settings and outcomes), but nonetheless the correlation is exclusively achieved by infinite-dimensional quantum systems. Our correlation exhibits, on slightly larger question and answer sets, a behaviour that was conjectured by Pál and Vértesi^10^ to be possessed by the *I*_3322_ Bell inequality^11^. While Pál and Vértesi gave strong numerical evidence for this behaviour^10^, an analytical proof has remained elusive.

In a related line of work, Slofstra^12^, and the subsequent^13–16^, provide non-local games which require arbitrarily high-dimensional strategies to attain arbitrarily close to optimal winning probabilities. However, for each of these games, any sequence of ideal strategies approaching the optimal winning probability does not have a well-defined limit, and the optimal correlation cannot be attained exactly (not even in infinite dimensions). Hence, the optimal correlations for such games separate ${{\mathcal{C}}}_{qs}$ from its closure, known as ${{\mathcal{C}}}_{qa}$, but do not shed any light on the relationship between ${{\mathcal{C}}}_{q}$ and ${{\mathcal{C}}}_{qs}$.

## Results

### An overview of the proof of separation

We start with an overview of the structure of the proof of our main result. To explain the argument, we start by giving an idealized version that runs against a barrier, and then talk about how to avoid the barrier.

Inspired by techniques from the field of device-independent self-testing (in particular refs. ^17,18^), which studies the certification of quantum states and measurements based solely on the observed correlations (see for example^19^ for a recent review on the topic), we will design a correlation p^*^ which guarantees the following two structural properties on the state attaining it: there is a local unitary Φ = Φ_*A*_ ⊗ Φ_*B*_ and an auxiliary state $\left|{\mathrm{aux}}\right\rangle$ such that

2 $$\Phi (\left|\psi \right\rangle )=\frac{1}{\sqrt{1+{\alpha }^{2}}}(\left|00\right\rangle +\alpha \left|11\right\rangle )\otimes \left|\mathrm{aux}\right\rangle .$$

Intuitively, the existence of such a local unitary says that the quantum system shared by Alice and Bob can be decomposed, up to local changes of basis, into a pair of entangled qubits (i.e. two-level systems) tensored with a (potentially entangled) state on an auxiliary system (of a priori unknown dimension). Second, there is a local unitary $\Phi ^{\prime}$ and an auxiliary state $\left|{\mathrm{aux}}^{\prime} \right\rangle$ such that

3 $$\Phi ^{\prime} (\left|\psi \right\rangle )=\left|\phi \right\rangle \oplus \frac{1}{\sqrt{1+{\alpha }^{2}}}(\left|00\right\rangle +\alpha \left|11\right\rangle )\otimes \left|\mathrm{aux}^{\prime} \right\rangle ,$$

where  ⊕  denotes a direct sum (and is meant to emphasize that the two summands have orthogonal supports).

Now, imagine we knew that the state $\left|\phi \right\rangle$ was separable, i.e., has Schmidt rank 1. Then, suppose towards a contradiction that $\left|\psi \right\rangle$ were finite-dimensional. Since Schmidt coefficients are preserved under local unitaries, from the first condition we see that the Schmidt rank of the state is even, while from the second condition we see that the Schmidt rank of the state is odd; contradiction.

In the above, the “magic” happens when we assume that $\left|\phi \right\rangle$ is separable. In general, any correlation that is attained using a separable $\left|\phi \right\rangle$ could also be attained by tensoring with extra entanglement and not making use of it in the measurements, so we will not be able to assume that $\left|\phi \right\rangle$ is separable. A different way of arguing about the set of Schmidt coefficients of $\left|\psi \right\rangle$ is required. In our main argument, our correlation p^*^ will still guarantee that $\left|\psi \right\rangle$ decomposes into two ways as in Eqs. (2) and (3), except that $\left|\phi \right\rangle$ is not necessarily separable. In place of the odd/even constraints, we perform a more fine-grained analysis of the self-testing guarantees enforced by our correlation p^*^. This allows us to obtain a stricter characterization of the set of Schmidt coefficients of the state $\left|\psi \right\rangle$, and of how the coefficients in Eqs. (2) and (3) relate to each other. In particular, we show that the decompositions Eqs. (2) and (3) partition the set of Schmidt coefficients of $\left|\psi \right\rangle$ into two different ways so that the set of nonzero Schmidt coefficients is in 1-to-1 correspondence with a proper subset of itself. Of course, this can only happen if the set of Schmidt coefficients is infinite.

### Correlations and basic self-tests

Given sets ${\mathcal{X}},{\mathcal{Y}}$, ${\mathcal{A}}$, ${\mathcal{B}}$, a (bipartite) correlation is a collection $\{p(a,b| x,y):a\in {\mathcal{A}}, b\in {\mathcal{B}}\}_{(x,y)\in {\mathcal{X}}\times {\mathcal{Y}}}$, where each *p*( ⋅ , ⋅ ∣*x*, *y*) is a probability distibution over ${\mathcal{A}}\times {\mathcal{B}}$. We interpret the correlation as describing the outcomes of a measurement scenario with two parties, say Alice and Bob. *p*(*a*, *b*∣*x*, *y*) is the probability that Alice outputs *a* and Bob outputs *b*, given that Alice used measurement setting *x* and Bob used setting *y*. ${\mathcal{X}}$ and ${\mathcal{Y}}$ are referred to as the question sets, while ${\mathcal{A}}$ and ${\mathcal{B}}$ are referred to as as the answer sets.

Given question sets and answer sets ${\mathcal{X}}$, ${\mathcal{Y}}$, ${\mathcal{A}}$, ${\mathcal{B}}$, a quantum strategy is specified by Hilbert spaces ${{\mathcal{H}}}_{A}$ and ${{\mathcal{H}}}_{B}$, a pure state $\left|\psi \right\rangle \in {{\mathcal{H}}}_{A}\otimes {{\mathcal{H}}}_{B}$, and projective measurements ${\{{\Pi }_{{A}_{x}}^{a}\}}_{a}$ on ${{\mathcal{H}}}_{A}$, ${\{{\Pi }_{{B}_{y}}^{b}\}}_{b}$ on ${{\mathcal{H}}}_{B}$, for $x\in {\mathcal{X}},y\in {\mathcal{Y}}$. We say that it induces correlation *p* if, for all $a\in {\mathcal{A}},b\in {\mathcal{B}},x\in {\mathcal{X}},y\in {\mathcal{Y}}$.

4 $$p(a,b| x,y)=\left\langle \psi | {\Pi }_{{A}_{x}}^{a}\otimes {\Pi }_{{B}_{y}}^{b}| \psi \right\rangle$$

Certain correlations have the special property that they are attained by a unique quantum strategy, up to local isometries. In this case, we say that the correlation “self-tests” the quantum strategy.

We introduce the tilted CHSH inequality^20^, a building block for the separating correlation that appears in this work. First, we recall the more well-known CHSH inequality^2^. It states that for binary observables *A*_0_, *A*_1_ on Hilbert space ${{\mathcal{H}}}_{A}$ and binary observables *B*_0_, *B*_1_ on Hilbert space ${{\mathcal{H}}}_{B}$ together with a product state $\left|\phi \right\rangle =\left|{\phi }_{A}\right\rangle \otimes \left|{\phi }_{B}\right\rangle$, we have

5 $$\langle \phi | {A}_{0}{B}_{0}+{A}_{0}{B}_{1}+{A}_{1}{B}_{0}-{A}_{1}{B}_{1}| \phi \rangle \le 2,$$

where the maximum is achieved (for example setting all observables to identity). However, if instead of the product state $\left|\phi \right\rangle$ we allow an entangled state $\left|\psi \right\rangle$, then the right-hand side of the inequality increases to $2\sqrt{2}$. This maximum requires a maximally entangled pair of qubits (EPR pair) to attain. In other words, a correlation attaining the maximum self tests an EPR pair. In this work, we would like to use an inequality that self tests a nonmaximally entangled state to attain the maximum; this is the tilted CHSH inequality. Given a real parameter *β* ∈ [0, 2], for a product state $\left|\phi \right\rangle =\left|{\phi }_{A}\right\rangle \otimes \left|{\phi }_{B}\right\rangle$,

6 $$\langle \phi | \beta {A}_{0}+{A}_{0}{B}_{0}+{A}_{0}{B}_{1}+{A}_{1}{B}_{0}-{A}_{1}{B}_{1}| \phi \rangle \le 2+\beta .$$

For entangled $\left|\psi \right\rangle$, we have instead that

7 $$\langle \psi | \beta {A}_{0}+{A}_{0}{B}_{0}+{A}_{0}{B}_{1}+{A}_{1}{B}_{0}-{A}_{1}{B}_{1}| \psi \rangle \le \sqrt{8+2{\beta }^{2}}.$$

It is known^21,22^ that the RHS of Eq. (7) is attained by a unique strategy, up to local isometries, in which Alice and Bob share the tilted EPR pair $\cos \theta (\left|00\right\rangle +\alpha \left|11\right\rangle )$, where $\alpha =\tan \theta$ and $\sin 2\theta =\sqrt{\frac{4\,-\,{\beta }^{2}}{4\,+\,{\beta }^{2}}}$.

Since in the present work we are primarily concerned with the ratio of the coefficients of the ideal state, we refer to the correlation attaining the RHS of Eq. (7) as the ideal tilted CHSH correlation for ratio *α*.

A convenient way to describe correlations is through correlation tables. A correlation *p* on ${\mathcal{X}}$, ${\mathcal{Y}}$, ${\mathcal{A}}$, ${\mathcal{B}}$ is completely specified by correlation tables *T*_*xy*_ for $x\in {\mathcal{X}},y\in {\mathcal{Y}}$, with entries *T*_*xy*_(*a*, *b*) = *p*(*a*, *b*∣*x*, *y*). See Table 1.

**Table 1:** The correlation table on question (*x*, *y*) of a correlation on answer sets ${\mathcal{A}}={\mathcal{B}}=\{0,1\}$.

*a\b*	0	1
**0**	*p*(0, 0∣*x*, *y*)	*p*(0, 1∣*x*, *y*)
**1**	*p*(1, 0∣*x*, *y*)	*p*(1, 1∣*x*, *y*)

As mentioned earlier, we will make use of the ideal tilted CHSH correlation as a building block for our separating correlation. For *x*, *y* ∈ {0, 1} and *α* ∈ (0, 1), we denote by ${{\rm{CHSH}}}_{x,y}^{\alpha }$ the correlation table on question *x*, *y* for the ideal tilted CHSH correlation for ratio *α*.

### The separating correlation

In this section, we describe the correlation p^*^ that separates ${{\mathcal{C}}}_{q}$ and ${{\mathcal{C}}}_{qs}$. The correlation is on question sets ${\mathcal{X}}=\{0,1,2,3\}$ and ${\mathcal{Y}}=\{0,1,2,3,4\}$ and answer sets ${\mathcal{A}}={\mathcal{B}}=\{0,1,2\}$. In this section, we introduce p^*^ by describing the ideal infinite-dimensional strategy that attains it. Our description here is at a high level, and we refer the reader to Supplementary Note 3 for the full detail. In the following section, we will prove that no finite-dimensional strategy attains p^*^.

Alice and Bob start with the infinite-dimensional bipartite entangled state

8 $$\left|\Psi \right\rangle =\sqrt{1-{\alpha }^{2}}\mathop{\sum }\limits_{i = 0}^{\infty }{\alpha }^{i}\left|ii\right\rangle \ ,$$

which may be thought of as an entangled state of two infinite-level systems.

They measure their half of $\left|\Psi \right\rangle$ as follows:

(i) For questions *x*, *y* ∈ {0, 1}, they each decompose their register into a direct sum of 2 × 2 blocks and perform the ideal tilted CHSH measurements for ratio *α* on each block.
(ii) For *x*, *y* ∈ {2, 3}, they do the same, but with a block structure which is shifted forward by one standard basis element. To be more explicit, in (i) the 2 × 2 blocks are spanned by pairs of basis elements of the form $(\left|2m\right\rangle ,\left|2m+1\right\rangle )$, while here they are spanned by pairs of the form $(\left|2m+1\right\rangle ,\left|2m+2\right\rangle )$. Notice that this is possible because the ratio between any two consecutive coefficients is *α*.
(iii) In addition, Bob has a fifth question (*y* = 4) on which he performs the same measurement as Alice performs on question *x* = 0.

Now, consider a state $\left|\psi \right\rangle$ and some measurements that reproduce the correlations above. Intuitively, (i) forces $\left|\psi \right\rangle$ to be (up to a local isometry) of the form

9 $$\left|\psi \right\rangle \approx \frac{1}{\sqrt{1+{\alpha }^{2}}}(\left|00\right\rangle +\alpha \left|11\right\rangle )\otimes \left|\mathrm{aux}\right\rangle ,$$

while (ii) forces $\left|\psi \right\rangle$ to be of the form

10 $$\left|\psi \right\rangle \approx \left|\phi \right\rangle \oplus \frac{1}{\sqrt{1+{\alpha }^{2}}}(\left|00\right\rangle +\alpha \left|11\right\rangle )\otimes \left|\mathrm{aux}^{\prime} \right\rangle ,$$

for some state $\left|\phi \right\rangle$. The correlations in (iii) serve the purpose of relating the two decompositions Eqs. (9) and (10). This allows us, for example, to argue that the set of Schmidt coefficients from the term $\alpha \left|11\right\rangle \otimes \left|\mathrm{aux}\right\rangle$ in Eq. (9) is exactly equal to the set of Schmidt coefficients from the term $\left|00\right\rangle \otimes \left|\mathrm{aux}^{\prime} \right\rangle$ in Eq. (10).

The ideal state and measurements defining p^*^ specify correlation tables *T*_*xy*_ for all pairs of questions *x* ∈ {0, 1, 2, 3}, *y* ∈ {0, 1, 2, 3, 4}. We explicitly report some of them, as we will later make use of the relations that these impose on the measurement projectors. For ease of notation let $C=\frac{1}{1-{\alpha }^{2}}$ in the tables below (note *C* > 1).

Formally, our main result is the following.

### Theorem 1

The correlation p^*^ *described above is not attained by any finite-dimensional strategy.*

Since, by definition, p^*^ is attained by the infinite-dimensional strategy above, this implies:

### Corollary 1

$${{\mathcal{C}}}_{q}\;\ne\;{{\mathcal{C}}}_{qs}$$.

The next section illustrates the main ideas in the proof of Theorem 1. We will first infer necessary properties of any state and measurements which attain the ideal correlation p^*^. Then, we characterize the set of Schmidt coefficients of such a state, and conclude that the set must be countably infinite.

### Forcing infinitely many Schmidt coefficients

For the rest of this section, let $(\left|\psi \right\rangle \in {{\mathcal{H}}}_{A}\otimes {{\mathcal{H}}}_{B},\{{\Pi }_{{A}_{x}}^{a}\},\{{\Pi }_{{B}_{y}}^{b}\})$ be a strategy attaining the ideal correlation p^*^ from section “The separating correlation”. As we have mentioned earlier, the structure of the ideal correlation p^*^ imposes a very special form on the state $\left|\psi \right\rangle$.

More precisely, the (weighted) ideal tilted CHSH correlations contained in Tables 2 and 3 imply, by the self-testing properties of the tilted CHSH inequality combined with a technical lemma about direct sums of correlations (we refer to Supplementary Note 4 for full details), that there exist local isometries Φ, $\Phi ^{\prime}$ and (normalized) auxiliary states $\left|{\mathrm{aux}}\right\rangle ,\left|\mathrm{aux}^{\prime} \right\rangle ,\left|\mathrm{aux}^{\prime\prime} \right\rangle$ such that

$$	{\rm{(i)}}\ \ \Phi (|\psi \rangle )\approx \, (| 00 \rangle +\alpha | 11\rangle )\otimes | {\mathrm{aux}}\rangle \\ 	{\rm{(ii)}}\ \ \Phi^{\prime} (|\psi \rangle ) \approx \, | 22 \rangle \otimes |{\mathrm{aux}}^{\prime\prime} \rangle \\ 	\, \oplus \eta ( |11\rangle +\alpha | 00 \rangle )\otimes | {\mathrm{aux}}^{\prime} \rangle$$

where $$C=\frac{1}{1\;-\;{\alpha }^{2}}$$ and $$\eta =\frac{\sqrt{C\;-\;1}}{\sqrt{1\;+\;{\alpha }^{2}}}$$.

**Table 2:** *T*_*xy*_ for *x*, *y* ∈ {0, 1}.

*a\b*	0	1	2
**0**	CHSH${}_{x,y}^{\alpha }$		0
**1**			0
**2**	0	0	0

The top-left 2 × 2 block contains ideal tilted CHSH correlations for questions *x*, *y*.

**Table 3:** *T*_*xy*_ for *x*, *y* ∈ {2, 3}.

*a\b*	1	0	2
**1**	$\frac{C-1}{C}\cdot {{\rm{CHSH}}}_{x,y}^{\alpha }$		0
**0**			0
**2**	0	0	$\frac{1}{C}$

Let $\bar{x},\bar{y}$ be *x*, *y* modulo 2. The top-left 2 × 2 block contains the ideal tilted CHSH correlation table for questions $\bar{x},\bar{y}$, weighted by $\frac{C-1}{C}$ (notice that we have flipped the 0 and 1 labels in the rows and columns).

As we have argued in the overview of section “An overview of the proof of separation”, the two decompositions (i) and (ii) are not enough to carry out an argument based solely on the parity of the set of Schmidt coefficients in (i) and (ii). This is because $\left|{\mathrm{aux}}^{\prime\prime} \right\rangle$ need not be a separable state.

We overcome this difficulty through Bob’s fifth question (*y* = 4). The correlation Tables 4 and 5, involving Bob’s fifth question, allow us to relate the Schmidt coefficients of (i) and (ii). More precisely, let Φ, $\Phi ^{\prime}$ and $\left|\mathrm{aux}\right\rangle$, $\left|\mathrm{aux}^{\prime} \right\rangle$, $\left|\mathrm{aux}^{\prime\prime} \right\rangle$ be as in (i) and (ii). Recall that the set of Schmidt coefficients of a state is preserved under local isometries. What we obtain is that the Schmidt coefficients from the even and odd terms respectively in (i) and (ii) must match up: the multiset of Schmidt coefficients from the $\left|00\right\rangle \otimes \left|\mathrm{aux}\right\rangle$ term in (i) (call this *S*_0_) equals the union of the multisets of Schmidt coefficients from the terms $\left|22\right\rangle \otimes \left|\mathrm{aux}^{\prime\prime} \right\rangle$ (call this *S*_2_) and $\eta \alpha \left|00\right\rangle \otimes \left|\mathrm{aux}^{\prime} \right\rangle$ (which is hence *S*_0_⧹*S*_2_) in (ii); the multiset of Schmidt coefficients from the $\alpha \left|11\right\rangle \otimes \left|\mathrm{aux}\right\rangle$ term in (i) (call this *S*_1_) equals the multiset from the term $\eta \left|11\right\rangle \otimes \left|\mathrm{aux}^{\prime} \right\rangle$ in (ii) (which is hence also *S*_1_). For a detailed proof of such relations we refer to Lemma 10 in Supplementary Note 4.

**Table 4:** *T*_*xy*_ for *x* = 0, *y* = 4.

*a\b*	0	1	2
**0**	$\frac{1}{C}\cdot \frac{1}{1\;-\;{\alpha }^{4}}$	0	0
**1**	0	$\frac{1}{C}\cdot \frac{{\alpha }^{2}}{1\;-\;{\alpha }^{4}}$	0
**2**	0	0	0

**Table 5:** *T*_*xy*_ for *x* = 2, *y* = 4.

*a\b*	0	1	2
**0**	$\frac{1}{C}\cdot (\frac{1}{1\;-\;{\alpha }^{4}}-1)$	0	0
**1**	0	$\frac{1}{C}\cdot \frac{{\alpha }^{2}}{1\;-\;{\alpha }^{4}}$	0
**2**	$\frac{1}{C}$	0	0

The proof that the set of Schmidt coefficients of $\left|\psi \right\rangle$ is countably infinite is completed by the following observations.

First notice that the set *S*_0_ is in bijection with *S*_1_, where the bijection is *f*: *S*_0_ → *S*_1_ such that *f*(*λ*)  = *αλ*. Second, we also have that *S*_1_ is in bijection with *S*_0_⧹*S*_2_ where the bijection is *g*: *S*_1_ → *S*_0_⧹*S*_2_ such that *g*(*λ*) = *αλ*. Finally, composing the maps *f* and *g* yields a bijection between *S*_0_ and *S*_0_⧹*S*_2_. Since *S*_2_ is nonempty, this implies that *S*_0_ must be infinite.

## Discussion

Understanding the relationship between known models of entanglement is a goal of fundamental theoretical importance, with potential repercussions on practical experiments.

Recent work has seen important advances in this direction, with a sequence of two breakthrough works by Slofstra^23,24^ showing first that the tensor product model (commonly adopted in quantum information) and the commuting-operator model (commonly adopted in algebraic quantum field theory) give rise to different sets of correlations, and later refining this to show that the set of correlations in the tensor product model is not equal to its closure.

Such works have also shown that answering foundational questions about the theory of entanglement is not only of theoretical significance, but brings forward important insights that result in potential applications in quantum information protocols, for example in the certification of high-dimensional entanglement^16,25,26^ or high-dimensional states and measurements^27^.

The work of Slofstra has left two main open questions. The first asks whether the commuting-operator model is stricly more powerful than the closure of the tensor product model. This question is known to be equivalent to Connes’ embedding conjecture^28^, a major-open problem in the mathematical field of operator algebras. The second asks whether infinite-dimensional quantum correlations in the tensor product model are strictly more powerful than their finite-dimensional counterpart. In this work, we answer the latter question in the affirmative, by giving the first example of an inherently infinite-dimensional quantum correlation. This is a correlation on just five questions per party, and three answers per party, which is only attained exactly by infinite-dimensional systems.

At first sight it appears that our correlation provides a test that can tell apart an infinite-dimensional system from a finite-dimensional one, and hence, in principle, a test that can assert whether nature allows the existence of systems with infinitely many degrees of freedom. However, this is not the case: although our correlation can only be exactly attained by two-entangled infinite-dimensional systems, for example two-entangled systems with infinite energy levels, it can be approximated arbitrarily well by systems of high enough, but finite, dimension, or in other words, by projecting onto subspaces of bounded energy. Thus, no experiment (which can only estimate statistics to a finite precision) can tell the two cases apart. This is not a shortcoming of our separating correlation, but rather a fundamental limitation that stems from the fact that the sets ${{\mathcal{C}}}_{q}$ and ${{\mathcal{C}}}_{qs}$ possess the same closure^29^. It is striking that we observe such a fundamental theoretical difference between finite and infinite-dimensional models of entanglement, yet we are inherently limited in our ability to distinguish the two models by the finiteness of the data we can gather.

A natural direction for future work is to investigate what are the smallest question and answer set sizes that witness such separation. The (3, 3, 2, 2) scenario is the simplest one that is suspected to separate ${{\mathcal{C}}}_{q}$ and ${{\mathcal{C}}}_{qs}$. Numerical evidence about the *I*_3322_ inequality^10^ suggests that the infinite-dimensional ideal measurements achieving the conjectured maximal violation have a block-diagonal form, with similarities to the form of our ideal measurements. This suggests that the study of this inequality is potentially amenable to ideas and techniques from our work.

## Supplementary information

Supplementary Information

## References

[1] Bell JS (1964). On the einstein podolsky rosen paradox. Physics.

[2] Clauser JF, Horne MA, Shimony A, Holt RA (1969). Proposed experiment to test local hidden-variable theories. Phys. Rev. Lett..

[3] Brunner N (2008). Testing the dimension of hilbert spaces. Phys. Rev. Lett..

[4] Cerf NJ, Bourennane M, Karlsson A, Gisin N (2002). Security of quantum key distribution using d-level systems. Phys. Rev. Lett..

[5] Ahrens J, Badziag P, Cabello A, Bourennane M (2012). Experimental device-independent tests of classical and quantum dimensions. Nat. Phys..

[6] Huber M, Pawłowski M (2013). Weak randomness in device-independent quantum key distribution and the advantage of using high-dimensional entanglement. Phys. Rev. A.

[7] Krenn, M. et al. Generation and confirmation of a (100 × 100)-dimensional entangled quantum system. *Proc. Natl. Acad. Sci. USA* **111**, 6243–6247 (2014). 10.1073/pnas.1402365111 PMC4035950 24706902

[8] Bavaresco J, Valencia NH, Klöckl C (2018). Measurements in two bases are sufficient for certifying high-dimensional entanglement. Nat. Phys..

[9] Tsirelson BS (1993). Some results and problems on quantum bell-type inequalities. Hadronic J. Suppl..

[10] Pál KF, Vértesi T (2010). Maximal violation of a bipartite three-setting, two-outcome bell inequality using infinite-dimensional quantum systems. Phys. Rev. A.

[11] Froissart M (1981). Constructive generalization of bellas inequalities. Il Nuovo Cim. B (1971-1996).

[12] Slofstra, W. The set of quantum correlations is not closed. *Forum Mathematics, Pi* **7**, e1 (2019).

[13] Dykema, K., Paulsen, V. I. & Prakash, J. Non-closure of the set of quantum correlations via graphs. *Commun*. *Math. Phys*. **365**, 1125–1142 (2019).

[14] Musat, M. & Rørdam, M. Non-closure of quantum correlation matrices and factorizable channels that require infinite dimensional ancilla. Preprint at http://arxiv.org/abs/1806.10242 (2018).

[15] Ji, Z.Leung, D. & Vidick, T. A three-player coherent state embezzlement game. Preprint at http://arxiv.org/abs/1802.04926 (2018).

[16] Coladangelo A (2020). two-player dimension witness based on embezzlement, and an elementary proof of the non-closure of the set of quantum correlations. Quantum.

[17] Coladangelo A, Goh KT, Scarani V (2017). All pure bipartite entangled states can be self-tested. Nat. Commun..

[18] Coladangelo A (2018). Generalization of the clauser-horne-shimony-holt inequality self-testing maximally entangled states of any local dimension. Phys. Rev. A.

[19] Šupić, I. & Bowles, J. Self-testing of quantum systems: a review. Preprint at http://arxiv.org/abs/1904.10042 (2019).

[20] Acín A, Massar S, Pironio S (2012). Randomness versus nonlocality and entanglement. Phys. Rev. Lett..

[21] Yang TH, Navascués M (2013). Robust self-testing of unknown quantum systems into any entangled two-qubit states. Phys. Rev. A.

[22] Bamps C, Pironio S (2015). Sum-of-squares decompositions for a family of clauser-horne-shimony-holt-like inequalities and their application to self-testing. Phys. Rev. A.

[23] Slofstra W (2020). Tsirelson’s problem and an embedding theorem for groups arising from non-local games. Journal of the American Mathematical Society.

[24] Slofstra, W. The set of quantum correlations is not closed. *Forum Mathematics, Pi* **7**, e1 (2019).

[25] Slofstra, W. & Vidick, T. Entanglement in non-local games and the hyperlinear profile of groups. *Annales Henri Poincaré* **19**, 2979–3005 (2018).

[26] Slofstra, W. A group with at least subexponential hyperlinear profile. Preprint at http://arxiv.org/abs/1806.05267 (2018).

[27] Coladangelo, A. & Stark, J. Robust self-testing for linear constraint system games. Preprint at http://arxiv.org/abs/1709.09267 (2017).

[28] Ozawa N (2013). About the connes embedding conjecture. Jpn. J. Math..

[29] Scholz, V. B. & Werner, R. F. Tsirelson’s problem. *P*reprint at http://arxiv.org/abs/0812.4305 (2008).

