# Supplementary material for: An inherently infinite-dimensional quantum correlation
Source: Nat Commun. 2020 Jul 3;11:3335. doi: 10.1038/s41467-020-17077-9 (PMC7335085; doi:10.1038/s41467-020-17077-9)
Supplement: Supplementary file 1 — Supplementary Information [file 41467_2020_17077_MOESM1_ESM.pdf]

Supplementary Information - An inherently infinite-dimensional quantum correlation

Andrea Coladangelo and Jalex Stark

Supplementary Notes

Supplementary Note 1 - Notation and preliminaries

For a positive integer n, we denote by $[n]$ the set $\{1, .., n\}$. δ_{ij} is the Kronecker delta. For a Hilbert space $\mathcal{H}$, $\mathcal{L}(\mathcal{H})$ is the space of linear operators on $\mathcal{H}$. Define the Pauli matrices

$$\sigma^z = \begin{pmatrix} 1 & 0 \\ 0 & -1 \end{pmatrix}, \text{ and } \sigma^x = \begin{pmatrix} 0 & 1 \\ 1 & 0 \end{pmatrix}. \tag{1}$$

For an operator $T \in \mathcal{L}(\mathcal{H})$ and a subspace $\mathcal{H}' \subseteq \mathcal{H}$ invariant under T, we denote by $T|_{\mathcal{H}'} \in \mathcal{L}(\mathcal{H}')$ the restriction of T to $\mathcal{H}'$. Let $\mathbb{C}^{\mathbb{N}}$ denote the Hilbert space of square-summable sequences, sometimes called $\ell^2(\mathbb{C})$. We endow it with a standard basis $\{|i\rangle : i \in \mathbb{N}\}$. Formally, $\mathbb{C}^{\mathbb{N}} = \left\{\sum_i a_i |i\rangle : \sum_i \left|a_i^2\right| < \infty\right\}$.

Correlations and quantum strategies were introduced in the main text. Here, we clarify some additional terminology.

We will sometimes refer to a quantum strategy as a triple $\left(|\psi\rangle, \{\Pi^a_{A_x}\}_a, \{\Pi^b_{B_y}\}_b\right)$. If we wish to emphasize the underlying Hilbert space, we write $\left(|\psi\rangle \in \mathcal{H}_A \otimes \mathcal{H}_B, \{\Pi^a_{A_x}\}_a, \{\Pi^b_{B_y}\}_b\right)$. Notice that we have chosen our state to be pure and our measurements to be projective. This choice is without loss of generality. The most general measurements are modeled by POVMs, but Naimark's dilation theorem implies that any correlation induced using POVMs can also be induced using projective measurements (possibly of larger dimension). Likewise, any correlation induced by a mixed state can also be induced by using a purification of that state. We sometimes describe a quantum strategy by specifying an observable for each question. The observables in turn specify the projectors through their eigenspaces.

A correlation is said to be quantum if there exists a quantum strategy that induces it. We refine this, and we say that a quantum correlation is *finite-dimensional* (*infinite-dimensional*) if it is induced by a quantum strategy on a finite-dimensional (infinite-dimensional) Hilbert space. We denote by $\mathcal{C}_q^{m,n,r,s}$ and $\mathcal{C}_{qs}^{m,n,r,s}$ respectively the sets of finite and infinite-dimensional quantum correlations on question sets of sizes m, n and answer sets of sizes r, s.

As we will be borrowing inspiration and techniques from the field of device-independent self-testing, we provide a formal definition:

Definition 1 (Self-testing)**.** *We say that a correlation $\{p^*(a,b|x,y) : a \in \mathcal{A}, b \in \mathcal{B}\}_{x\in\mathcal{X},y\in\mathcal{Y}}$ self-tests a strategy $\left(|\Psi\rangle, \{\tilde{\Pi}^a_{A_x}\}_a, \{\tilde{\Pi}^b_{B_y}\}_b\right)$ if, for any strategy $\left(|\psi\rangle, \{\Pi^a_{A_x}\}_a, \{\Pi^b_{B_y}\}_b\right)$ that induces p^*, there exists a local isometry $\Phi = \Phi_A \otimes \Phi_B$ and an auxiliary state $|aux\rangle$ such that, for all $x \in \mathcal{X}, y \in \mathcal{Y}, a \in \mathcal{A}, b \in \mathcal{B}$,*

$$\Phi(|\psi\rangle) = |\Psi\rangle \otimes |aux\rangle \tag{2}$$

$$\Phi(\Pi^a_{A_x} \otimes \Pi^b_{B_y} |\psi\rangle) = \tilde{\Pi}^a_{A_x} \otimes \tilde{\Pi}^b_{B_y} |\Psi\rangle \otimes |aux\rangle \tag{3}$$

Sometimes, we refer to *self-testing of the state* when we are only concerned with the guarantee of Supplementary Equation (2), and not Supplementary Equation (3).

The tilted CHSH inequality was introduced in the main text. Here, we report the ideal strategy that attains maximal violation of the inequality, as we will employ these ideal measurements as part of of the ideal strategy defining our separating correlation.

The maximum in the tilted CHSH inequality is attained by the following strategy:

Definition 2 (Ideal strategy for tilted CHSH)**.** *Given parameter β, let $\sin 2\theta = \sqrt{\frac{4-\beta^2}{4+\beta^2}}$, $\mu = \arctan \sin 2\theta$, and $\alpha = \tan\theta$. Define the α-*tilted Pauli operators *as*

$$\sigma^z_\alpha := \cos\mu\sigma^z + \sin\mu\sigma^x, \text{ and } \sigma^x_\alpha := \cos\mu\sigma^z - \sin\mu\sigma^x. \tag{4}$$

The ideal strategy for tilted CHSH with parameter β (i.e. achieving the RHS of Equation (7) from the main text) consists of the joint state $|\Psi\rangle = \cos\theta(|00\rangle + \alpha|11\rangle)$ and observables A_0, A_1 and B_0, B_1

with $A_0 = \sigma^z$, $A_1 = \sigma^x$, $B_0 = \sigma^z_\alpha$ and $B_1 = \sigma^x_\alpha$. For each observable, we associate the projection onto the $+1$-eigenspace with answer 0 and the projection onto the -1-eigenspace with answer 1.

We will make use of the following lemma.

Lemma 3 ([1]). *The tilted CHSH correlation for ratio α self-tests the strategy of Definition 2.*

Supplementary Note 2 - Direct sums of correlations

In this section, we introduce the notion of a direct sum of correlations. We will later use this to build our desired correlation out of tilted CHSH building blocks. Lemma 5 will allow us to characterize the strategies for the desired correlation from self-testing results about its direct summands. In particular, these strategies also decompose, in a sense made precise below, as a direct sum of strategies corresponding to the direct summands. The proof is somewhat technical, and the ideas in the proof are not necessary to understand the rest of the proof. Some of the ideas in the proof have appeared ad-hoc in previous works on constructing quantum correlations block-by-block [3], [2]. We have packaged these arguments into a lemma since it may be of independent interest. First, we define formally a direct sum of correlations. For $\omega \in [0,1]$ and a correlation table T_{xy}, we write $\omega \cdot T_{xy}$ to denote entry-wise multiplication of T_{xy} by ω. We may refer to ω as a *weight*.

Definition 4 (Direct sum of correlations). *Let p be a correlation on $\mathcal{X}, \mathcal{Y}, \mathcal{A}, \mathcal{B}$. Suppose for some positive integer l, for $i \in [l]$, there exist partitions $\mathcal{A} = \bigsqcup_{i=1}^{l} \mathcal{A}_i$, $\mathcal{B} = \bigsqcup_{i=1}^{l} \mathcal{B}_i$, real numbers $\omega_i \geq 0$ with $\sum_{i=1}^{l} \omega_i = 1$, and correlations p_i on $\mathcal{X}, \mathcal{Y}, \mathcal{A}_i, \mathcal{B}_i$ such that for all $i, j \in [l]$, $a \in \mathcal{A}_i, b \in \mathcal{B}_j, x \in \mathcal{X}, y \in \mathcal{Y}$,*

$$p(a, b|x, y) = \delta_{ij}\omega_i p_i(a, b|x, y). \tag{5}$$

Then we say that p is a direct sum of the p_i, and we write $p = \oplus_{i=1}^{l}\omega_i p_i$. We sometimes refer to the p_i as blocks *of p and the ω_i as* weights *of the blocks. We give a visual interpretation of condition (5) in Supplementary Table 1.*

Supplementary Table 1: The correlation table for $p = \oplus_i \omega_i p_i$ on questions x, y. $T^{(i)}_{xy}$ is the correlation table for correlation p_i on questions x, y.

a \ b	$\mathcal{B}_1$	$\cdots$	$\mathcal{B}_l$
$\mathcal{A}_1$	$\omega_1 \cdot T^{(1)}_{xy}$	0	0
$\vdots$	0	$\ddots$	0
$\mathcal{A}_l$	0	0	$\omega_l \cdot T^{(l)}_{xy}$

Lemma 5. *Let $p \in \mathcal{C}_{qs}^{m,n,d,d}$ be a correlation on $\mathcal{X}, \mathcal{Y}, \mathcal{A}, \mathcal{B}$, induced by a strategy $(|\psi\rangle \in \mathcal{H}_A \otimes \mathcal{H}_B, \{\Pi^a_{A_x}\}_a, \{\Pi^b_{B_y}\}_b)$. Suppose for some positive integer l, there exist partitions $\mathcal{A} = \bigsqcup_{i=1}^{l} \mathcal{A}_i$, $\mathcal{B} = \bigsqcup_{i=1}^{l} \mathcal{B}_i$, with $|\mathcal{A}_i| = |\mathcal{B}_i| = d_i$, and correlations $p_i \in \mathcal{C}_{qs}^{m,n,d_i,d_i}$ on $\mathcal{X}, \mathcal{Y}, \mathcal{A}_i, \mathcal{B}_i$ such that $p = \bigoplus_{i=1}^{l} \omega_i p_i$. Then there exist direct sum decompositions $\mathcal{H}_A = \mathcal{H}_A^{null} \oplus \bigoplus_i \mathcal{H}_A^i$, $\mathcal{H}_B = \mathcal{H}_B^{null} \oplus \bigoplus_i \mathcal{H}_B^i$ and strategies*

$$\left(\frac{|\psi_i\rangle}{\||\psi_i\rangle\|} \in \mathcal{H}_A^i \otimes \mathcal{H}_B^i, \{\Pi^a_{A_x}|_{\mathcal{H}_A^i}\}_{a \in \mathcal{A}_i}, \{\Pi^b_{B_y}|_{\mathcal{H}_B^i}\}_{b \in \mathcal{B}_i} \right) \tag{6}$$

such that:

(i) Strategy (6) is well-defined, i.e. the restricted operators $\Pi^a_{\mathcal{A}_x}|_{\mathcal{H}^i_A}$ *and* $\Pi^b_{\mathcal{A}_y}|_{\mathcal{H}^i_B}$ *are projections.*

(ii) $\| \ket{\psi_i} \|^2 = \omega_i$.

(iii) p_i *is induced by strategy (6).*

(iv) For all $x \in \mathcal{X}, y \in \mathcal{Y}, a \in \mathcal{A}_i, b \in \mathcal{B}_i$*:*

$$\Pi^a_{A_x}|_{\mathcal{H}^i_A} \ket{\psi_i} = \Pi^a_{A_x} \ket{\psi} , \quad \Pi^B_{B_y}|_{\mathcal{H}^i_B} \ket{\psi_i} = \Pi^b_{B_y} \ket{\psi} . \tag{7}$$

Proof. For the remainder of the proof, when an operator acts only on one tensor factor we omit writing the identity on the other factors.

Our first goal is to construct the subspaces $\mathcal{H}^i_A, \mathcal{H}^i_B$. We first study the action of the projectors corresponding to answers in $\mathcal{A}_i$ and $\mathcal{B}_i$ on the state $\ket{\psi}$. We will use these properties to define the states $\ket{\psi_i}$. Then from these, we will construct $\mathcal{H}^i_A$ and $\mathcal{H}^i_B$.

For $x \in \mathcal{X}, y \in \mathcal{Y}$, define $\Pi^{\mathcal{A}_i}_{A_x} := \sum_{a \in \mathcal{A}_i} \Pi^a_{A_x}$ and $\Pi^{\mathcal{B}_i}_{B_y} := \sum_{b \in \mathcal{B}_i} \Pi^b_{B_y}$. We will show that $\Pi^{\mathcal{A}_i}_{A_x} \ket{\psi} = \Pi^{\mathcal{B}_i}_{B_y} \ket{\psi}$ for all i, x, y. For any $i \in [l], x \in \mathcal{X}, y \in \mathcal{Y}$,

$$\begin{aligned}
\Pi^{\mathcal{A}_i}_{A_x} \ket{\psi} &= \left(\sum_{a \in \mathcal{A}_i} \Pi^a_{A_x} \right) \otimes I \ket{\psi} && (8)\\
&= \left(\sum_{a \in \mathcal{A}_i} \Pi^a_{A_x} \right) \otimes \left(\sum_{b \in \mathcal{B}} \Pi^b_{B_y} \right) \ket{\psi} && (9)\\
&= \left(\sum_{a \in \mathcal{A}_i} \Pi^a_{A_x} \right) \otimes \left(\sum_{b \in \mathcal{B}_i} \Pi^b_{B_y} \right) \ket{\psi} && (10)\\
&= \Pi^{\mathcal{A}_i}_{A_x} \otimes \Pi^{\mathcal{B}_i}_{B_y} \ket{\psi} . && (11)
\end{aligned}$$

The second equality follows from the fact that $\left\{ \Pi^b_{B_y} \right\}$ forms a complete measurement. The third equality comes from the block structure of the correlation. More specifically, suppose that $a \in \mathcal{A}_i$ but $b \notin \mathcal{B}_i$. Then the block structure demands that $p(a, b|x, y) = 0$ for all x, y. So we conclude that $\left\| \Pi^a_{A_x} \otimes \Pi^b_{B_y} \ket{\psi} \right\|^2 = p(a, b|x, y) = 0$. This forces the appropriate terms of the sum in Equation (9) to vanish. The same argument with the roles of $\mathcal{A}$ and $\mathcal{B}$ reversed gives

$$\Pi^{\mathcal{B}_i}_{B_y} \ket{\psi} = \Pi^{\mathcal{A}_i}_{A_x} \otimes \Pi^{\mathcal{B}_i}_{B_y} \ket{\psi} . \tag{12}$$

Combined with Equation (11), this implies that, for any i, x, y,

$$\Pi^{\mathcal{A}_i}_{A_x} \ket{\psi} = \Pi^{\mathcal{B}_i}_{B_y} \ket{\psi} \tag{13}$$

In particular, the action of $\Pi^{\mathcal{A}_i}_{A_x}$ on $\ket{\psi}$ is the same for all x, and similarly for the $\mathcal{B}$ operators. This lets us define

$$\ket{\psi_i} := \Pi^{\mathcal{A}_i}_{A_x} \ket{\psi} , \tag{14}$$

where the choice of x does not matter.

Now we compute the norm of $\ket{\psi_i}$. The block structure $p = \oplus_i \omega_i p_i$ of the correlation gives us that

for any fixed x and y,

$$\omega_i = \sum_{a\in\mathcal{A}_i, b\in\mathcal{B}_i} p(a,b|x,y) \tag{15}$$

$$= \sum_{a\in\mathcal{A}_i, b\in\mathcal{B}_i} \left\langle \psi \,\middle|\, \Pi^a_{A_x} \otimes \Pi^b_{B_y} \,\middle|\, \psi \right\rangle \tag{16}$$

$$= \left\langle \psi \,\middle|\, \Pi^{\mathcal{A}_i}_{A_x} \otimes \Pi^{\mathcal{B}_i}_{B_y} \,\middle|\, \psi \right\rangle \tag{17}$$

$$= \| |\psi_i\rangle \|^2 . \tag{18}$$

where the last line follows from Supplementary Equation (13). This establishes condition (ii). Now let $\rho^i_A = \mathrm{Tr}_B |\psi_i\rangle\langle\psi_i| = \sum_j \lambda_j |j\rangle\langle j|$, where λ_j are the eigenvalues and $|j\rangle$ the eigenvectors of ρ^i_A. These are guaranteed to exist even if $|\psi_i\rangle$ is infinite-dimensional, because the existence of a Schmidt decomposition for any bipartite state holds also in infinite-dimensional Hilbert spaces. Notice that

$$\sum_j \lambda_j = \mathrm{Tr}\, \rho^i_A = \| |\psi_i\rangle \|^2 = \omega_i. \tag{19}$$

We wish to compute the action of $\Pi^{\mathcal{A}_i}_{A_x}$ on the eigenstates of ρ^i_A. We calculate

$$\omega_i = \left\langle \psi \,\middle|\, \Pi^{\mathcal{A}_i}_{A_x} \otimes I \,\middle|\, \psi \right\rangle \tag{20}$$

$$= \mathrm{Tr}\, \Pi^{\mathcal{A}_i}_{A_x} \rho^i_A \tag{21}$$

$$= \sum_j \lambda_j \,\mathrm{Tr}\, \Pi^{\mathcal{A}_i}_{A_x} |j\rangle\langle j| \tag{22}$$

$$= \sum_j \lambda_j \left\| \Pi^{\mathcal{A}_i}_{A_x} |j\rangle \right\|^2 . \tag{23}$$

Since $\omega_i = \sum_j \lambda_j$, we must have $\left\| \Pi^{\mathcal{A}_i}_{A_x} |j\rangle \right\|^2 = 1$ for each j. In other words, $\Pi^{\mathcal{A}_i}_{A_x} |j\rangle = |j\rangle$. This motivates us to define the space $\mathcal{H}^i_A$ as the span of the nontrivial eigenvectors of ρ^i_A. Define also P_i as the projection onto subspace $\mathcal{H}^i_A$.

It follows from the definition of the $|\psi_i\rangle$ and the $\mathcal{H}^i_A$ that

$$P_i |\psi_j\rangle = \delta_{ij} |\psi_i\rangle . \tag{24}$$

Furthermore, notice that $\Pi^{\mathcal{A}_i}_{A_x} P_i = P_i$. Thus the $\mathcal{H}^i_A$ are suitable spaces for the new strategies to be defined on. In particular, the restricted operators $\Pi^a_{A_x}|_{\mathcal{H}^i_A}$ are projectors. To see this, notice that they are orthogonal for distinct a and that they sum to identity.

Let $\mathcal{H}^{\mathrm{null}}_A$ be the orthogonal complement of $\bigoplus_i \mathcal{H}^i_A$ in $\mathcal{H}_A$. Define $\mathcal{H}^i_B$ and $\mathcal{H}^{\mathrm{null}}_B$ analogously. Clearly, $\bigoplus_i \mathcal{H}^i_A$ and $\bigoplus_i \mathcal{H}^i_B$ are topologically closed. This implies that $\mathcal{H}_A = \mathcal{H}^{\mathrm{null}}_A \oplus \bigoplus_i \mathcal{H}^i_A$ and $\mathcal{H}_B = \mathcal{H}^{\mathrm{null}}_B \oplus \bigoplus_i \mathcal{H}^i_B$.

Thus, we have established condition (i) of the lemma.

It follows straightforwardly from the Definition of $|\psi_i\rangle$ and (24) that for $a \in \mathcal{A}_i$, $\Pi^a_{A_x}|_{\mathcal{H}^i_A} |\psi_i\rangle = \Pi^a_{A_x} |\psi\rangle$, and similarly for $\mathcal{B}$. This establishes condition (iv). Finally, we show condition (iii), that the strategies in each block induce the appropriate correlations. We fix arbitrary $a \in \mathcal{A}_i, b \in \mathcal{B}_i, x \in \mathcal{X}, y \in \mathcal{Y}$, and calculate

$$\frac{1}{\| |\psi_i\rangle \|^2} \left\langle \psi_i \middle| \Pi^a_{A_x}|_{\mathcal{H}^i_A} \otimes \Pi^b_{B_y}|_{\mathcal{H}^i_B} \middle| \psi_i \right\rangle = \frac{1}{\omega_i} \left\langle \psi \,\middle|\, \Pi^a_{A_x} \otimes \Pi^b_{B_y} \,\middle|\, \psi \right\rangle \tag{25}$$

$$= \frac{1}{\omega_i} p(a,b|x,y) \tag{26}$$

$$= p_i(a,b|x,y). \tag{27}$$

In the above, the first quantity is the correlation induced by the strategy defined in Equation (6), and the last quantity is the desired correlation p_i. Thus, we have shown condition (iii).

□

Supplementary Note 3 - The separating correlation

In this section, we describe in detail the correlation p^* that separates $\mathcal{C}_q$ and $\mathcal{C}_{qs}$, which was described only at a high-level in the main text.

The correlation is on question sets $\mathcal{X} = \{0,1,2,3\}$ and $\mathcal{Y} = \{0,1,2,3,4\}$ and answer sets $\mathcal{A} = \mathcal{B} = \{0,1,2\}$. Hence, the smallest classes we separate are $\mathcal{C}_q^{4,5,3,3}$ and $\mathcal{C}_{qs}^{4,5,3,3}$. We define p^* by describing the ideal infinite-dimensional strategy that induces it. In the following section, we will prove that no finite-dimensional strategy induces p^*.

Recall the definition of $\mathbb{C}^{\mathbb{N}}$ from Supplementary Note 1. For each $m \geq 0$, we define two isometries $V_m^{\text{even}}, V_m^{\text{odd}} : \mathbb{C}^2 \to \mathbb{C}^{\mathbb{N}}$ as follows:

$$V_m^{\text{even}} |0\rangle = |2m\rangle, V_m^{\text{even}} |1\rangle = |2m+1\rangle, \text{ and } V_m^{\text{odd}} |0\rangle = |2m+1\rangle, V_m^{\text{odd}} |1\rangle = |2m+2\rangle. \quad (28)$$

We use these isometries to define observables on $\mathbb{C}^{\mathbb{N}}$. By abuse of notation, for an isometry $V : \mathbb{C}^2 \to C^{\mathbb{N}}$ and an operator O on $\mathbb{C}^2$, we write $V(O)$ to refer to the pushforward $VOV^\dagger$ of O along V. For example, $V_m^{\text{even}}(\sigma^z) = |2m\rangle\langle 2m| - |2m+1\rangle\langle 2m+1|$. For O an operator with $+1, 0, -1$ eigenvalues, we write O^+ for the projection onto the $+1$ eigenspace and O^- for the projection onto the -1 eigenspace. One can check that with this notation $O = O^+ - O^-$. We use the notation $\bigoplus A_i$ to denote the direct sum of observables A_i. We will make use of the α-tilted Paulis $\sigma_\alpha^z, \sigma_\alpha^x$ from Definition 2. The following is the ideal strategy in detail.

Definition 6 (Ideal state and measurements for $p^* \in \mathcal{C}_{qs}^{4,5,3,3}$)**.** *Fix $\alpha \in (0,1)$. The correlation $p^* \in \mathcal{C}_{qs}^{4,5,3,3}$ is specified by the quantum strategy $(|\Psi\rangle \in \mathbb{C}^{\mathbb{N}} \otimes \mathbb{C}^{\mathbb{N}}, \{\Pi_{A_x}^a\}_a, \{\Pi_{B_y}^b\}_b)$, where $|\Psi\rangle = \sqrt{1-\alpha^2}\sum_{i=0}^\infty \alpha^i |ii\rangle$, and the ideal measurements are described in Supplementary Tables 2 and 3.*

Supplementary Table 2: Alice's ideal measurements. The entry in cell x, a is the projector $\Pi_{A_x}^a$.

x \ a	0	1	2
0	$[\bigoplus_{m=0}^\infty V_m^{\text{even}}(\sigma^z)]^+$	$[\bigoplus_{m=0}^\infty V_m^{\text{even}}(\sigma^z)]^-$	0
1	$[\bigoplus_{m=0}^\infty V_m^{\text{even}}(\sigma^x)]^+$	$[\bigoplus_{m=0}^\infty V_m^{\text{even}}(\sigma^x)]^-$	0
2	$[\bigoplus_{m=0}^\infty V_m^{\text{odd}}(\sigma^z)]^-$	$[\bigoplus_{m=0}^\infty V_m^{\text{odd}}(\sigma^z)]^+$	$\|0\rangle\langle 0\|$
3	$[\bigoplus_{m=0}^\infty V_m^{\text{odd}}(\sigma^x)]^-$	$[\bigoplus_{m=0}^\infty V_m^{\text{odd}}(\sigma^x)]^+$	$\|0\rangle\langle 0\|$

Supplementary Table 3: Bob's ideal measurements. The entry in cell y, b is the projector $\Pi^b_{B_y}$.

y \ b	0	1	2
0	$[\bigoplus_{m=0}^{\infty} V_m^{\text{even}}(\sigma_\alpha^z)]^+$	$[\bigoplus_{m=0}^{\infty} V_m^{\text{even}}(\sigma_\alpha^z)]^-$	0
1	$[\bigoplus_{m=0}^{\infty} V_m^{\text{even}}(\sigma_\alpha^x)]^+$	$[\bigoplus_{m=0}^{\infty} V_m^{\text{even}}(\sigma_\alpha^x)]^-$	0
2	$[\bigoplus_{m=0}^{\infty} V_m^{\text{odd}}(\sigma_\alpha^z)]^-$	$[\bigoplus_{m=0}^{\infty} V_m^{\text{odd}}(\sigma_\alpha^z)]^+$	$\|0\rangle\langle 0\|$
3	$[\bigoplus_{m=0}^{\infty} V_m^{\text{odd}}(\sigma_\alpha^x)]^-$	$[\bigoplus_{m=0}^{\infty} V_m^{\text{odd}}(\sigma_\alpha^x)]^+$	$\|0\rangle\langle 0\|$
4	$[\bigoplus_{m=0}^{\infty} V_m^{\text{even}}(\sigma^z)]^+$	$[\bigoplus_{m=0}^{\infty} V_m^{\text{even}}(\sigma^z)]^-$	0

See the main text, for an informal intuition about the ideal measurements above.

The ideal state and measurements defining p^* specify correlation tables T_{xy} for all pairs of questions $x \in \{0,1,2,3\}$, $y \in \{0,1,2,3,4\}$. We report some of them here for convenience (they also appear in the main text), as we will later analyse the constraints that these impose on the measurement projectors. Let $C = \frac{1}{1-\alpha^2}$ in the tables below (note $C > 1$). Recall from the main text that we denote by $\text{CHSH}^\alpha_{x,y}$ the correlation table on question $x, y \in \{0,1\}$ for the ideal tilted CHSH correlation for ratio α.

Supplementary Table 4: On the left, T_{xy} for $x, y \in \{0,1\}$. The top-left 2×2 block contains ideal tilted CHSH correlations for questions x, y.

a \ b	0	1	2
0	$\text{CHSH}^\alpha_{x,y}$		0
1			0
2	0	0	0

a \ b	1	0	2
1	$\frac{C-1}{C} \cdot \text{CHSH}^\alpha_{\bar{x},\bar{y}}$		0
0			0
2	0	0	$\frac{1}{C}$

Supplementary Table 5: On the right, T_{xy} for $x, y \in \{2,3\}$. Let $\bar{x}, \bar{y}$ be x, y modulo 2. The top-left 2×2 block contains the ideal tilted CHSH correlation table for questions $\bar{x}, \bar{y}$, weighted by $\frac{C-1}{C}$ (notice that we have flipped the 0 and 1 labels in the rows and columns.)

Supplementary Table 6: On the left, T_{xy} for $x = 0, y = 4$

a \ b	0	1	2
0	$\frac{1}{C} \cdot \frac{1}{1-\alpha^4}$	0	0
1	0	$\frac{1}{C} \cdot \frac{\alpha^2}{1-\alpha^4}$	0
2	0	0	0

a \ b	0	1	2
0	$\frac{1}{C} \cdot (\frac{1}{1-\alpha^4} - 1)$	0	0
1	0	$\frac{1}{C} \cdot \frac{\alpha^2}{1-\alpha^4}$	0
2	$\frac{1}{C}$	0	0

Supplementary Table 7: On the right, T_{xy} for $x = 2, y = 4$

Supplementary Note 4 - Proof of separation

In this section, we give a detailed proof of Theorem 1 from the main text. We start from a strategy that induces p^*: in Supplementary Note 4.1, we prove properties of the state and the measurement operators, and in Supplementary Note 4.2, we characterize the non-zero Schmidt coefficients of the state, concluding that they must be infinitely many.

Supplementary Note 4.1 - Characterizing the state and the projectors

The following lemma establishes the existence of two local isometries which decompose any state achieving p^* into two different ways (as anticipated in the proof overview of Section 2 from the main text).

Lemma 7 (Characterizing the state and projectors)**.** *Let* $(|\psi\rangle \in \mathcal{H}_A \otimes \mathcal{H}_B, \{\Pi^a_{A_x}\}, \{\Pi^b_{B_y}\})$ *be a strategy inducing the ideal correlation* p^* *from Definition 6. Let* $C = \frac{1}{1-\alpha^2}$*. Then there exist two local isometries* Φ *and* Φ' *and (normalized) states* $|aux\rangle$*,* $|aux'\rangle$ *and* $|aux''\rangle$ *such that*

(i)
- $\Phi(|\psi\rangle) = \frac{1}{\sqrt{1+\alpha^2}}(|00\rangle + \alpha\,|11\rangle) \otimes |aux\rangle$
- $\Phi(\Pi^0_{A_0} \otimes I\,|\psi\rangle) = \frac{1}{\sqrt{1+\alpha^2}}\,|00\rangle \otimes |aux\rangle$
- $\Phi(\Pi^1_{A_0} \otimes I\,|\psi\rangle) = \frac{\alpha}{\sqrt{1+\alpha^2}}\,|11\rangle \otimes |aux\rangle$

(ii)
- $\Phi'(|\psi\rangle) = \frac{1}{\sqrt{C}}\,|22\rangle \otimes |aux''\rangle \oplus \sqrt{\frac{C-1}{C}}\frac{1}{\sqrt{1+\alpha^2}}(|11\rangle + \alpha\,|00\rangle) \otimes |aux'\rangle$
- $\Phi'(\Pi^0_{A_2} \otimes I\,|\psi\rangle) = \sqrt{\frac{C-1}{C}}\frac{\alpha}{\sqrt{1+\alpha^2}}\,|00\rangle \otimes |aux'\rangle$
- $\Phi'(\Pi^1_{A_2} \otimes I\,|\psi\rangle) = \sqrt{\frac{C-1}{C}}\frac{1}{\sqrt{1+\alpha^2}}\,|11\rangle \otimes |aux'\rangle$
- $\Phi'(\Pi^2_{A_2} \otimes I\,|\psi\rangle) = \frac{1}{\sqrt{C}}\,|22\rangle \otimes |aux''\rangle$

Proof. (i): Let p' be the restriction of p^* to questions $x, y \in \{0,1\}$. From Table 4, we know that p' is the ideal tilted CHSH correlation for ratio α (except that it has an extra answer "2" which has zero probability mass). Applying the block decomposition lemma (Lemma 5) with $\omega_1 = 1$ and $\omega_2 = 0$, we have that there exist subspaces $\mathcal{H}^1_A \subseteq \mathcal{H}_A$ and $\mathcal{H}^1_B \subseteq \mathcal{H}_B$ such that the strategy $(|\psi\rangle \in \mathcal{H}^1_A \otimes \mathcal{H}^1_B, \{\Pi^a_{A_x}|_{\mathcal{H}^1_A}\}_{a\in\{0,1\}}, \{\Pi^b_{B_y}|_{\mathcal{H}^1_B}\}_{b\in\{0,1\}})$ induces the ideal tilted CHSH correlation.

By Lemma 3, the tilted CHSH correlation self-tests its ideal strategy, i.e. there exists a local isometry $\Phi_1 = \Phi_{1,A} \otimes \Phi_{1,B}$ with $\Phi_{1,A} : \mathcal{H}^1_A \to \tilde{\mathcal{H}}^1_A \otimes \tilde{\mathcal{H}}^1_{A,aux}$ and $\Phi_{1,B} : \mathcal{H}^1_B \to \tilde{\mathcal{H}}^1_B \otimes \tilde{\mathcal{H}}^1_{B,aux}$, and a (normalized) state $|aux\rangle \in \tilde{\mathcal{H}}^1_{A,aux} \otimes \tilde{\mathcal{H}}^1_{B,aux}$ such that $\Phi_1(|\psi\rangle) = \frac{1}{\sqrt{1+\alpha^2}}(|00\rangle + \alpha\,|11\rangle) \otimes |aux\rangle$. Moreover, by Lemma 3, it is also the case that

$$\Phi_1\left((\Pi^0_{A_0}|_{\mathcal{H}^1_A} - \Pi^1_{A_0}|_{\mathcal{H}^1_A}) \otimes I\,|\psi\rangle\right) = Z \otimes I \frac{1}{\sqrt{1+\alpha^2}}(|00\rangle + \alpha\,|11\rangle) \otimes |aux\rangle\,. \tag{29}$$

Since $(I+Z)/2 = |0\rangle\langle 0|$ and $(I-Z)/2 = |1\rangle\langle 1|$, we deduce by linearity that

$$\Phi_1\left(\Pi^0_{A_0}|_{\mathcal{H}^1_A} \otimes I\,|\psi\rangle\right) = \frac{1}{\sqrt{1+\alpha^2}}\,|00\rangle \otimes |aux\rangle \;\text{ and }\; \Phi_1\left(\Pi^1_{A_0}|_{\mathcal{H}^1_A} \otimes I\,|\psi\rangle\right) = \frac{\alpha}{\sqrt{1+\alpha^2}}\,|11\rangle \otimes |aux\rangle\,. \tag{30}$$

Letting Φ be any isometric extension of Φ_1 to $\mathcal{H}_A \otimes \mathcal{H}_B$ and applying condition (iv) of Lemma 5 gives (i).

(ii): Let p'' be the restriction of p^* to questions $x, y \in \{2,3\}$. Then from table 5 we have that $p'' = \omega_1 p_1 \oplus \omega_2 p_2$ where p_1 is the ideal tilted CHSH correlation (for ratio α) and p_2 is the correlation in which answer $(2,2)$ has probability 1 on all question pairs, and $\omega_1 = \frac{C-1}{C}$, $\omega_2 = \frac{1}{C}$.

By Lemma 5, there exist subspaces $\mathcal{H}_A^{\text{null}}, \mathcal{H}_B^{\text{null}}$, $\mathcal{H}_A^1, \mathcal{H}_A^2$, $\mathcal{H}_B^1, \mathcal{H}_B^2$ with $\mathcal{H}_A = \mathcal{H}_A^{\text{null}} \oplus \mathcal{H}_A^1 \oplus \mathcal{H}_A^2$ and $\mathcal{H}_B = \mathcal{H}_B^{\text{null}} \oplus \mathcal{H}_B^1 \oplus \mathcal{H}_B^2$, and strategies S_1 and S_2 with

$$S_1 = \left(\frac{|\psi_1\rangle}{\| |\psi\rangle \|} \in \mathcal{H}_A^1 \otimes \mathcal{H}_B^1, \{\Pi_{A_x}^a|_{\mathcal{H}_A^1}\}_{a\in\{0,1\}}, \{\Pi_{B_y}^b|_{\mathcal{H}_B^1}\}_{b\in\{0,1\}} \right), \tag{31}$$

$$S_2 = \left(\frac{|\psi_2\rangle}{\| |\psi_2\rangle \|} \in \mathcal{H}_A^2 \otimes \mathcal{H}_B^2, \{\Pi_{A_x}^2|_{\mathcal{H}_A^2}\}, \{\Pi_{B_y}^2|_{\mathcal{H}_B^2}\} \right) \tag{32}$$

such that $\| |\psi_1\rangle \|^2 = \frac{C-1}{C}$, $\| |\psi_2\rangle \|^2 = \frac{1}{C}$ and $|\psi\rangle = |\psi_1\rangle + |\psi_2\rangle$. Moreover, S_1 induces the ideal tilted CHSH correlation for ratio α (with the roles of the 0 and 1 answers flipped — see Table 5). As in the proof of (i), we can apply Lemma 3 to obtain local isometries $\Phi_1 = \Phi_{1,A} \otimes \Phi_{1,B}$ with $\Phi_{1,A} : \mathcal{H}_A^1 \to \tilde{\mathcal{H}}_A^1 \otimes \tilde{\mathcal{H}}_{A,aux}^1$ and $\Phi_{1,B} : \mathcal{H}_B^1 \to \tilde{\mathcal{H}}_B^1 \otimes \tilde{\mathcal{H}}_{B,aux}^1$, and a (normalized) state $|aux'\rangle \in \tilde{\mathcal{H}}_{A,aux}^1 \otimes \tilde{\mathcal{H}}_{B,aux}^1$ such that

(a) $\Phi_1(|\psi_1\rangle) = \sqrt{\frac{C-1}{C}} \frac{1}{\sqrt{1+\alpha^2}} (|11\rangle + \alpha\, |00\rangle) \otimes |aux'\rangle$, (we have flipped the zero and one basis elements for later convenience)

(b) $\Phi_1(\Pi_{A_2}^1|_{\mathcal{H}_A^1} \otimes I\, |\psi_1\rangle) = \sqrt{\frac{C-1}{C}} \frac{1}{\sqrt{1+\alpha^2}} |11\rangle \otimes |aux'\rangle$, and

(c) $\Phi_1(\Pi_{A_2}^0|_{\mathcal{H}_A^1} \otimes I\, |\psi_1\rangle) = \sqrt{\frac{C-1}{C}} \frac{\alpha}{\sqrt{1+\alpha^2}} |00\rangle \otimes |aux'\rangle$.

where (b) and (c) are obtained similarly as in part (i) of this proof.

Now, let $\Phi_2 = \Phi_{2,A} \otimes \Phi_{2,B}$, with $\Phi_{2,A} : \mathcal{H}_A^2 \to \tilde{\mathcal{H}}_A^2 \otimes \tilde{\mathcal{H}}_{A,aux}^2$ and $\Phi_{2,B} : \mathcal{H}_B^2 \to \tilde{\mathcal{H}}_B^2 \otimes \tilde{\mathcal{H}}_{B,aux}^2$ be a local isometry, and $|aux''\rangle \in \tilde{\mathcal{H}}_{A,aux}^2 \otimes \tilde{\mathcal{H}}_{B,aux}^2$ a (normalized) state such that

(d) $\Phi_2(|\psi_2\rangle) = \frac{1}{\sqrt{C}} |22\rangle \otimes |aux''\rangle$.

Such Φ_2 and $|aux''\rangle$ trivially exist.

Define

- $\Phi'_A : \mathcal{H}_A^1 \oplus \mathcal{H}_A^2 \to (\tilde{\mathcal{H}}_A^{(1)} \otimes \tilde{\mathcal{H}}_{A,aux}^{(1)}) \oplus (\tilde{\mathcal{H}}_A^{(2)} \otimes \tilde{\mathcal{H}}_{A,aux}^{(2)})$ as $\Phi'_A = \Phi_{1,A} \oplus \Phi_{2,A}$
- $\Phi'_B : \mathcal{H}_B^1 \oplus \mathcal{H}_B^2 \to (\tilde{\mathcal{H}}_B^{(1)} \otimes \tilde{\mathcal{H}}_{B,aux}^{(1)}) \oplus (\tilde{\mathcal{H}}_B^{(2)} \otimes \tilde{\mathcal{H}}_{B,aux}^{(2)})$ as $\Phi'_B = \Phi_{1,B} \oplus \Phi_{2,B}$

Let Φ''_A be any isometric extension of Φ'_A to $\mathcal{H}_A$, and let Φ''_B be any isometric extension of Φ'_B to $\mathcal{H}'_B$. Let $\Phi' = \Phi''_A \otimes \Phi''_B$. Then (a), (b), (c) and (d), together with condition (iv) of Lemma 5, imply that Φ' satisfies condition (ii) of Lemma 7, as desired.

□

We also need the following properties, obtained using the $y = 4$ question on Bob's side.

Lemma 8. *Let $(|\psi\rangle, \{\Pi_{A_x}^a\}, \{\Pi_{B_y}^b\})$ be a strategy inducing p^*. The following properties hold:*

(i) $\Pi_{A_0}^0 |\psi\rangle = \Pi_{B_4}^0 |\psi\rangle = (\Pi_{A_2}^2 + \Pi_{A_2}^0) |\psi\rangle$

(ii) $\Pi_{A_0}^1 |\psi\rangle = \Pi_{B_4}^1 |\psi\rangle = \Pi_{A_2}^1 |\psi\rangle$

(iii) $|\psi\rangle = \Pi_{A_0}^0 \otimes \Pi_{B_4}^0 |\psi\rangle + \Pi_{A_0}^1 \otimes \Pi_{B_4}^1 |\psi\rangle$

Proof. From correlation table 6, we read out that $\langle\psi| \Pi_{A_0}^0 \Pi_{B_4}^0 |\psi\rangle = \|\Pi_{A_0}^0 |\psi\rangle \|^2 = \|\Pi_{B_4}^0 |\psi\rangle \|^2$. By the Cauchy-Schwarz inequality, this implies that $\Pi_{A_0}^0 |\psi\rangle = \Pi_{B_4}^0 |\psi\rangle$. Similarly, from correlation table 7, we deduce $(\Pi_{A_2}^2 + \Pi_{A_2}^0) |\psi\rangle = \Pi_{B_4}^0 |\psi\rangle$, which yields (i). We derive (ii) analogously. Item (iii) follows from combining the previous two items with the equality $(\Pi_{A_0}^0 + \Pi_{A_0}^1) |\psi\rangle = |\psi\rangle$. □

Supplementary Note 4.2 - Characterizing the Schmidt coefficients

From now onwards, let $(|\psi\rangle, \{\Pi^a_{A_x}\}, \{\Pi^b_{B_y}\})$ be any strategy inducing p^*. In the previous subsection, we gave a partial characterization of the operators and state . In this subsection, we make use of these properties to show that $|\psi\rangle$ has infinitely many non-zero Schmidt coefficients. For a bipartite state $|\phi\rangle_{AB}$, we denote by $\mathrm{Sch}(|\phi\rangle_{AB})$ the multiset of non-zero Schmidt coefficients of $|\phi\rangle_{AB}$ (here by multiset we mean a set with multiplicity, sometimes called an unordered list; for example, the multiset of Schmidt coefficients of the EPR pair is $(\frac{1}{\sqrt{2}}, \frac{1}{\sqrt{2}}))$. Recall that the Schmidt coefficients $\{\lambda_i\}$ are the unique nonnegative real numbers so that $|\phi\rangle_{AB} = \sum_i \lambda_i |i\rangle_A \otimes |i\rangle_B$ for some bases of the A and B registers. Any such pair of bases is called a pair of *Schmidt bases with respect to* $|\phi\rangle$. Usually the tensor product decomposition of the Hilbert space will be clear, in which case we'll simply write $\mathrm{Sch}(|\phi\rangle)$ without the subscripts. We will use the following basic fact about Schmidt coefficients; we provide a proof for completeness.

Lemma 9. *Let $|\psi\rangle, |\phi\rangle, |\eta\rangle$ be states on $\mathcal{H}_A \otimes \mathcal{H}_B$ with $|\psi\rangle = |\phi\rangle + |\eta\rangle$. Define reduced densities*

$$\rho_A = \mathrm{Tr}_B |\psi\rangle\langle\psi|, \sigma_A = \mathrm{Tr}_B |\phi\rangle\langle\phi|, \tau_A = \mathrm{Tr}_B |\eta\rangle\langle\eta| \tag{33}$$

on $\mathcal{H}_A$. Define ρ_B, σ_B, τ_B similarly. Suppose that $|\phi\rangle$ and $|\eta\rangle$ are "orthogonal on both subsystems" in the sense that $\sigma_A\tau_A = 0 = \sigma_B\tau_B$. Then $\mathrm{Sch}(|\psi\rangle) = \mathrm{Sch}(|\phi\rangle) \sqcup \mathrm{Sch}(|\eta\rangle)$, where $\sqcup$ denotes disjoint union.

Proof. A Schmidt basis for $\mathcal{H}_A$ with respect to $|\psi\rangle$ is the same as an eigenbasis for the reduced density operator $\mathrm{Tr}_B |\psi\rangle\langle\psi|$. Using the orthogonality of $|\phi\rangle$ and $|\eta\rangle$, one can check that the three densities ρ_A, σ_A, τ_A commute. Therefore, the densities have a common eigenbasis. This is also a common Schmidt basis. After repeating the argument to find a common Schmidt basis on $\mathcal{H}_B$, we can write the states as

$$|\psi\rangle = \sum_i \lambda_i |ii\rangle, |\phi\rangle = \sum_i a_i |ii\rangle, \text{ and } |\eta\rangle = \sum_i b_i |ii\rangle, \tag{34}$$

with $a_i + b_i = \lambda_i$. By the orthogonality of $|\eta\rangle$ and $|\phi\rangle$, we have $a_i b_i = 0$ for each i. This implies that for each i, exactly one of the following two equalities holds: $\lambda_i = a_i$ or $\lambda_i = b_i$. This yields the lemma. □

Lemma 10. *Let Φ, Φ' and $|aux\rangle$, $|aux'\rangle$, $|aux''\rangle$ be the local isometries and auxiliary states from Lemma 7. Let $S = \mathrm{Sch}(|\psi\rangle)$, and let $S_2 = \mathrm{Sch}\left(\frac{1}{\sqrt{C}} |22\rangle \otimes |aux''\rangle\right)$. Then there exists a partition $S = S_0 \sqcup S_1$ such that:*

- $S_0 = \mathrm{Sch}\left(\frac{1}{\sqrt{1+\alpha^2}} |00\rangle \otimes |aux\rangle\right) = S_2 \sqcup \mathrm{Sch}\left(\sqrt{\frac{C-1}{C}} \frac{\alpha}{\sqrt{1+\alpha^2}} |00\rangle \otimes |aux'\rangle\right)$
- $S_1 = \mathrm{Sch}\left(\frac{\alpha}{\sqrt{1+\alpha^2}} |11\rangle \otimes |aux\rangle\right) = \mathrm{Sch}\left(\sqrt{\frac{C-1}{C}} \frac{1}{\sqrt{1+\alpha^2}} |11\rangle \otimes |aux'\rangle\right)$

Notice that these two equalities give us two different correspondences between the Schmidt coefficients of $|aux\rangle$ and $|aux'\rangle$, where one involves multiplying by α and the other involves dividing by α.

Proof. Recall from Lemma 8 that $|\psi\rangle = \Pi^0_{A_0} \otimes \Pi^0_{B_4} |\psi\rangle + \Pi^1_{A_0} \otimes \Pi^1_{B_4} |\psi\rangle$. We deduce by Lemma 9 that S can be partitioned into two sets S_0 and S_1, where

$$S_0 = \mathrm{Sch}\left(\Pi^0_{A_0} |\psi\rangle\right) \text{ and } S_1 = \mathrm{Sch}\left(\Pi^1_{A_0} |\psi\rangle\right). \tag{35}$$

Since local isometries preserve Schmidt coefficients, $\Phi(|\psi\rangle)$, $\Phi'(|\psi\rangle)$ and $|\psi\rangle$ have the same set of Schmidt coefficients S. Moreover, Lemma 7 gives

$$\Phi(\Pi^0_{A_0} |\psi\rangle) = \frac{1}{\sqrt{1+\alpha^2}} |00\rangle \otimes |aux\rangle \text{ and } \Phi(\Pi^1_{A_0} |\psi\rangle) = \frac{\alpha}{\sqrt{1+\alpha^2}} |11\rangle \otimes |aux\rangle. \tag{36}$$

By direct substitution,

$$S_0 = \mathrm{Sch}\left(\frac{1}{\sqrt{1+\alpha^2}}\left|00\right\rangle \otimes \left|aux\right\rangle\right) \text{ and } S_1 = \mathrm{Sch}\left(\frac{\alpha}{\sqrt{1+\alpha^2}}\left|11\right\rangle \otimes \left|aux\right\rangle\right). \tag{37}$$

By Lemma 8, we also have $\Pi^0_{A_0}\left|\psi\right\rangle = (\Pi^2_{A_2} + \Pi^0_{A_2})\left|\psi\right\rangle$ and $\Pi^1_{A_0}\left|\psi\right\rangle = \Pi^1_{A_2}\left|\psi\right\rangle$. Moreover, from Lemma 7, we also have $\Phi'((\Pi^2_{A_2} + \Pi^0_{A_2})\left|\psi\right\rangle) = \frac{1}{\sqrt{C}}\left|22\right\rangle \otimes \left|aux''\right\rangle + \sqrt{\frac{C-1}{C}}\frac{\alpha}{\sqrt{1+\alpha^2}}\left|00\right\rangle \otimes \left|aux'\right\rangle$ and $\Phi'(\Pi^1_{A_2}\left|\psi\right\rangle) = \sqrt{\frac{C-1}{C}}\frac{1}{\sqrt{1+\alpha^2}}\left|11\right\rangle \otimes \left|aux'\right\rangle$. Then this implies

$$S_0 = \mathrm{Sch}\left(\frac{1}{\sqrt{C}}\left|22\right\rangle \otimes \left|aux''\right\rangle + \sqrt{\frac{C-1}{C}}\frac{\alpha}{\sqrt{1+\alpha^2}}\left|00\right\rangle \otimes \left|aux'\right\rangle\right) \tag{38}$$

$$= S_2 \sqcup \mathrm{Sch}\left(\sqrt{\frac{C-1}{C}}\frac{\alpha}{\sqrt{1+\alpha^2}}\left|00\right\rangle \otimes \left|aux'\right\rangle\right), \text{ and} \tag{39}$$

$$S_1 = \mathrm{Sch}\left(\sqrt{\frac{C-1}{C}}\frac{1}{\sqrt{1+\alpha^2}}\left|11\right\rangle \otimes \left|aux'\right\rangle\right). \tag{40}$$

Putting together Equations (35) through (40) gives the statement of the Lemma. □

Theorem 11. *Let p^* be the ideal correlation introduced in Definition 6. Let $(\left|\psi\right\rangle, \{\Pi^a_{A_x}\}, \{\Pi^b_{B_y}\})$ be any strategy inducing p^*. Then $\left|\psi\right\rangle$ has infinitely many non-zero Schmidt coefficients.*

Proof. Let $\left|aux\right\rangle, \left|aux'\right\rangle$, S_0, S_1 and S_2 be as in Lemma 10. Recall from Lemma 10 that

$$S_0 = \mathrm{Sch}\left(\frac{1}{\sqrt{1+\alpha^2}}\left|00\right\rangle \otimes \left|aux\right\rangle\right) \text{ and } S_1 = \mathrm{Sch}\left(\frac{\alpha}{\sqrt{1+\alpha^2}}\left|11\right\rangle \otimes \left|aux\right\rangle\right). \tag{41}$$

Then we can rewrite these sets as

$$S_0 = \left\{\frac{1}{\sqrt{1+\alpha^2}}\lambda : \lambda \in \mathrm{Sch}\left(\left|aux\right\rangle\right)\right\} \text{ and } S_1 = \left\{\frac{1}{\sqrt{1+\alpha^2}}\alpha\lambda : \lambda \in \mathrm{Sch}\left(\left|aux\right\rangle\right)\right\} \tag{42}$$

Notice that there is a bijection $f : S_0 \to S_1$ such that $f(\lambda) = \alpha\lambda$. Again from Lemma 10 we have

$$S_0 = S_2 \sqcup \mathrm{Sch}\left(\sqrt{\frac{C-1}{C}}\frac{\alpha}{\sqrt{1+\alpha^2}}\left|00\right\rangle \otimes \left|aux'\right\rangle\right) \text{ and } S_1 = \mathrm{Sch}\left(\sqrt{\frac{C-1}{C}}\frac{1}{\sqrt{1+\alpha^2}}\left|11\right\rangle \otimes \left|aux'\right\rangle\right). \tag{43}$$

Then we can rewrite $S_0 \setminus S_2$ and S_1 as

$$S_0 \backslash S_2 = \left\{\sqrt{\frac{C-1}{C}}\frac{\alpha}{\sqrt{1+\alpha^2}}\lambda : \lambda \in \mathrm{Sch}\left(\left|aux'\right\rangle\right)\right\} \text{ and } S_1 = \left\{\sqrt{\frac{C-1}{C}}\frac{1}{\sqrt{1+\alpha^2}}\lambda : \lambda \in \mathrm{Sch}\left(\left|aux'\right\rangle\right)\right\}. \tag{44}$$

Notice that there is a bijection $g : S_1 \to S_0 \setminus S_2$ such that $g(\lambda) = \alpha\lambda$.

Composing the maps f and g yields a bijection between S_0 and $S_0 \setminus S_2$. Since S_2 is nonempty, this implies that S_0 must be infinite. □

Supplementary References

[1] Cédric Bamps and Stefano Pironio. Sum-of-squares decompositions for a family of clauser-horne-shimony-holt-like inequalities and their application to self-testing. *Physical Review A*, 91(5):052111, 2015.

[2] Andrea Coladangelo. A generalization of the CHSH inequality self-testing maximally entangled states of any local dimension. *Preprint at http://arxiv.org/abs/1803.05904*, 2018.

[3] Andrea Coladangelo, Koon Tong Goh, and Valerio Scarani. All pure bipartite entangled states can be self-tested. *Nature Communications 8*, page 15485, 2017.
